# Clinical and Financial Impacts of Late Referral in Patients With Chronic Kidney Disease: A Narrative Review

**DOI:** 10.7759/cureus.99708

**Published:** 2025-12-20

**Authors:** Rana Abou Mrad, Mohamed A Salman

**Affiliations:** 1 Department of Nephrology, Mediclinic Airport Road Hospital, Abu Dhabi, ARE; 2 Department of Clinical Research and Evidence Generation, Trillium Sciences, Dubai, ARE

**Keywords:** chronic kidney disease, cost-effectiveness, healthcare costs, late referral, renal replacement therapy

## Abstract

Chronic kidney disease (CKD) is a worldwide burden on healthcare systems. As the disease severity progresses, CKD patients are at high risk of morbidity, mortality, and renal replacement therapy. The cost of care for these conditions is substantially high and increases as the disease progresses. Early referral to nephrology and appropriate management can potentially improve outcomes and reduce costs if implemented efficiently. This review aimed to identify the causes of late referral to nephrology specialists and their impact on CKD costs. Proposed implications and recommended actions were included to address late referrals and ultimately reduce care costs. A thorough literature review was conducted using MEDLINE, Embase, and Scopus databases, covering studies published up to December 2024 on the causes of late referral to nephrology and its impact on the costs of CKD care. The causes of late referral are classified into (i) patient-related, (ii) physician-related, and (iii) health system-related factors. The financial burden of CKD is discussed, including the overall costs of CKD management and the impact of late referral on costs. The review included peer-reviewed articles discussing the causes and outcomes of late referral to nephrology specialists. Pediatric studies, acute kidney injury (AKI) diagnosis, or societal cost studies were excluded. Late referral rates to nephrology specialists remain high worldwide, ranging from 30% to 52%. Multiple causes of late referrals have been identified. Patient-related factors included low awareness, socioeconomic barriers, comorbidities, and psychological factors. Physician-related factors included the lack of familiarity among non-nephrology specialists with the indications for nephrology care or referral. Healthcare system-related factors included lack of referral guidelines, limited access to nephrology specialists, insurance and coverage barriers, and poor coordination between primary care physicians and nephrologists. In addition, this review demonstrated the significant financial burden of CKD worldwide, and the financial impact of late referral further increases the costs of CKD care. Serious measures must be undertaken to ensure that referrals are made promptly when outcomes can be modified. In conclusion, early referral of patients with CKD to nephrology specialists could improve clinical outcomes and reduce the financial burden on healthcare systems. Standardized referral guidelines, unified screening tools, electronic medical records, and telemedicine could enhance early nephrology consultation.

## Introduction and background

Chronic kidney disease (CKD) is a universal health concern that is continually growing. It affects around 9.1% of the population worldwide and was responsible for 4.6% of global deaths in 2017 [1]. The most common causes of CKDs are diabetes mellitus and hypertension, which are highly prevalent across the globe [1-4]. The number of individuals with end-stage renal disease (ESRD) requiring renal replacement therapy (RRT) continues to increase, exceeding 5.3 million worldwide [1].

As patients progress through CKD stages, they develop extensive healthcare needs and require referral to specialized nephrology care. Patients reaching ESRD are at high risk of cardiovascular morbidity and mortality compared to the general population. This imposes a substantial burden on the healthcare system. Most healthcare systems worldwide focus on mitigating risk factors and comorbidities that accelerate progression [5]. In a meta-analysis of 37 studies estimating the healthcare and societal costs of CKD, the mean annual total healthcare costs per individual with CKD stages 1-3 ranged from $1,600 to $25,037. The cost per individual with CKD stages 4 and 5 ranged from $5,367 to $53,186, whereas ESRD costs ranged from $20,110 to $100,591 per individual [6]. The annual cost of dialysis per patient in the Canadian healthcare system ranges from $71,000 to $107,000, depending on the setting, and the initial cost of transplantation is estimated at around $100,000 [7].

These staggering figures highlight a critical public health challenge, with ongoing efforts focused on cost-effective interventions to reduce the economic and clinical impact of disease progression. The clinical benefits of early referral include proactive management of CKD-related complications, enabling optimized blood pressure control, timely treatment of anemia, and early intervention for metabolic disturbances [8]. They also include increased access to transplantation [9], reduced mortality, enhanced predialysis preparedness, and shortened hospital stay at dialysis initiation [8].

There is an ongoing debate about whether early vs. late referral to nephrology in the predialytic stages of CKD can alter prognosis and reduce costs. Late referral to nephrology specialists is a challenge in most societies, with an estimated incidence reaching 52% in France and 49% in Germany [10]. More recent studies show a prevalence of late referrals fluctuating at around 30%, with some variations between industrialized and developing countries [11]. Clinical practice guidelines recommend co-management with nephrology at CKD stage 3 [12-14]. However, a recent study from Denmark revealed that only 16% of patients with an eGFR <30 mL/min/1.73 m² and 35% of patients with eGFR <15 mL/min/1.73 m² were in contact with a nephrologist in the outpatient setting [15]. Given the rising global burden of CKD, the timing of referral to nephrology is a key modifiable factor that can influence clinical outcomes and the economic impact of CKD on healthcare systems.

This review aimed to demonstrate that timely referral to nephrology specialists or clinics significantly reduces CKD-related costs and positively impacts clinical outcomes. It also explores the causes of late referral of CKD patients, looking at factors from the patient, physician, and healthcare system perspectives. We described how late referrals impact the overall cost of CKD and RRT care. Ultimately, the goal is to propose practical, evidence-based policy recommendations to ensure earlier referrals, improve patient outcomes, and reduce healthcare costs.

## Review

Study design, objectives, and ethical considerations

This narrative review was designed to perform a comprehensive literature review of the existing literature on the causes, costs, and policy implications related to the late referral of patients with CKD to nephrology specialists. It aimed to identify factors associated with the late referral of CKD patients, understand the associated healthcare costs at various CKD stages, and propose implications that could aid in optimizing patient referral and reducing healthcare expenditures. This study did not involve primary data collection from human subjects; therefore, ethical approval was not required. All data were extracted from publicly available peer-reviewed publications.

Literature search strategy

A thorough literature search was conducted using electronic databases, including MEDLINE, Embase, and Scopus. The search included data published from inception till December 2024. The search terms included a combination of keywords along with Boolean operators (AND, OR), “nephrology,” “chronic kidney disease,” “kidney failure,” “end-stage renal disease,” “renal replacement therapy,” “dialysis,” “transplantation,” “early referral,” “late referral,” “consultation,” “timing,” “economic burden,” “cost,” “cost-effectiveness,” and “healthcare utilization.” Included studies were peer-reviewed articles published in English, including meta-analyses, systematic reviews, randomized controlled trials, retrospective and prospective observational studies, and physician surveys. All selected studies examined the causes and impact of late referral to nephrology for CKD patients, along with the associated healthcare costs of CKD management. Studies were excluded if they focused solely on pediatric populations (<18 years), acute kidney injury (AKI), or the societal costs of CKD. Given the heterogeneity of study designs, definitions of early vs. late referral, and reported outcomes, no quantitative meta-analysis of original data was performed. Instead, we conducted a narrative review of the literature to integrate findings across the included studies. Data from the included studies were extracted using a predefined template that captured study design, country or region, population characteristics, definition of early vs. late referral, key clinical outcomes (e.g., mortality, hospitalization, dialysis initiation), and cost- or resource-related outcomes, if available. We performed a qualitative, narrative synthesis to integrate findings from the included studies, highlighting areas of consistency, difference, and uncertainty.

Definition of late referral

The progressive nature of CKD leads to a gradual and irreversible decline in kidney function. Late referral plays a huge role in the accelerated progression to ESRD. Rapid progression of CKD is defined as a sustained decline in eGFR of more than 5 mL/min/1.73 m² per year [16]. Under these circumstances, late referrals may be unavoidable due to the aggressive nature of the underlying kidney disease [17,18]. Early prediction of the need for nephrology referral in these cases cannot be easily anticipated. However, CKD is typically a slowly progressive disease, often taking years or even decades to advance to ESRD [19]. Therefore, the majority of CKD patients are expected to have a window of opportunity for timely referral to a nephrology specialist.

Referral time can depend on multiple interplaying factors simultaneously. There were conflicting results in the literature due to differences in healthcare systems, referral criteria, and study designs. Some studies defined late referral based on time to dialysis initiation, while others used eGFR thresholds, leading to different conclusions [20]. These interacting factors contribute to heterogeneous study results, reinforcing the complexity of CKD referral patterns. Understanding these differences is essential for promptly interpreting the literature and developing evidence-based strategies to improve early nephrology referrals and optimize patient outcomes (Table 1).

**Table 1:** Definitions of early referral to nephrology specialists

Study	Study design	Early referral definition	Country
Kinchen et al., 2002​ [21]	Prospective cohort	First nephrologist evaluation >12 months before dialysis initiation	USA
Cass et al., 2002​ [9]	Retrospective cohort	Nephrologist referral >3-4 months before need for dialysis	Australia
Avorn et al., 2002​ [22]	Retrospective cohort	First nephrologist encounter >90 days before initiation of dialysis	USA
Kazmi et al., 2004 [23]	Prospective cohort	First nephrology visit ≥4 months before starting dialysis	USA
Kim et al., 2013​ [24]	Prospective cohort	First nephrologist consultation >1 year before dialysis, with predialysis education provided	South Korea
Hommel et al., 2012 [25]	Prospective cohort	Follow-up in renal unit >16 weeks before RRT initiation	Denmark
Lee et al., 2014 [26]	Prospective cohort	Referred to a nephrologist >1 year before dialysis initiation	Korea
Blunt et al., 2015​ [27]	Retrospective cohort	Nephrology referral >90 days before start of dialysis (patients starting dialysis more than 3 months after referral)	UK
Lee et al., 2016 [28]	Prospective cohort	Referred to a nephrologist >1 year before dialysis initiation	Korea
Lonnemann et al., 2016 [29]	Retrospective cohort	When nephrology care was present in the starting year, or initiated during the following 3 years	Germany
Dhanorkar et al., 2022​ [30]	Observational cohort	First encounter with a nephrologist >1 year before dialysis initiation (with dialysis education provided)	India
Haarhaus et al., 2023 [31]	Retrospective	Patients who had received nephrological care before starting dialysis	Romania
Cheng et al., 2025 [32]	Systematic review and meta-analysis	Variable definition of included resources	China

Risk factors of late referral

**Figure 1:**
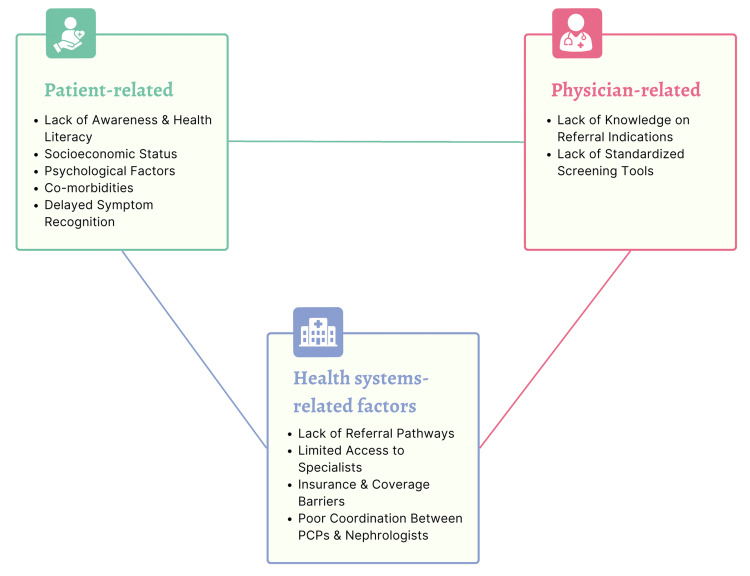
Summary of the main causes of referral delay in chronic kidney disease. PCPs: primary care physicians

Previous studies have identified multiple factors contributing to referral delay in CKD, which are generally categorized into the following three main groups: (i) patient-related factors, (ii) physician-related factors, and (iii) health system-related factors [33-40]. Figure 1 highlights the main causes of late referral across the three levels. It is important to differentiate between modifiable and non-modifiable factors associated with late referrals to nephrology specialists. Addressing modifiable barriers can improve the early referral rate to nephrology, eventually improving patient outcomes and decreasing the expected burden of the disease on the economy.

Patient-related factors

Patient Demographics

Age: Age has been identified as an independent factor influencing referral time. Previous studies indicated that elderly patients are at higher risk of referral delay [37,39,41-43]. Schwenger et al. reported that the median referral-to-dialysis interval was significantly shorter in elderly patients (3.5 weeks) compared to younger patients (20.5 weeks, p=0.007) [43]. Campbell et al. reported that age was considered an independent risk factor for decreased odds of referral to a nephrology specialist [42]. Another Korean study found that younger ages were associated with early referral to nephrology [28]. Hyperacute referral at the time of dialysis initiation was more common in older and more frail patients [11]. On the contrary, a previous study found that younger age was associated with late referral; however, this study used a lower age cutoff of 30 years [36]. Other studies have found no significant association between age and late referral, highlighting the variability in findings across different healthcare settings [35,40].

Gender and race: The same debate was evident regarding the impact of gender and race on the referral of CKD patients. Previous studies reported that female gender and non-White races are more likely to be referred [28,41,42]. In contrast, a European study found that women were less likely to visit a nephrologist, regardless of their condition or referral criteria [44]. Other studies showed no association between timing of referral and gender or race [39,40,45,46]. Some studies also reported race (African Americans or Hispanics) as a contributing factor for late referral [47], but other studies showed no similar association [36].

Socioeconomic Status

There is evidence that low socioeconomic status negatively impacts late referral [35]. Lee et al. showed that a low level of education, a low degree of family support, and male patients working in physically demanding professions, such as farming, fishing, laboring, and mechanics, were associated with shorter referral times [28]. In addition, patients who had a history of alcoholism, substance abuse, and homelessness/unemployment were found to be referred late to nephrology, less than one month before the onset of dialysis [48].

Psychological Factors

Upon informing CKD patients of their diagnosis and the potential need for RRT, psychological factors play a major role in their acceptance, compliance, and presentation for treatment [49,50]. As a result, many patients struggle to psychologically cope with the diagnosis, leading to denial or avoidance behaviors [51]. Fear and denial are the most notable culprits [10], with denial accounting for 45% of late referral cases among 460 patients of low economic status at a tertiary urban center in the United States [48]. Other psychological disorders, such as depression and psychosis, were identified as critical risk factors linked to the non-referral of CKD patients to nephrology specialists [52]. Predialysis psychoeducation significantly extended the time to RRT and improved survival benefits. However, the same study found no survival benefits for early referral compared to late referral [53].

Comorbidities

Most studies have linked the presence of comorbid conditions to late referral to nephrology [21,28,46,54]. Patients with other illnesses at the time of diagnosis of renal disease, mainly malignancies, heart failure, hypertension, and diabetes mellitus, were twice as likely to be referred late to nephrology than patients with no comorbidities [47]. Similar findings were reported in Korea, where patients with congestive heart failure and limited mobility were also found to have late referrals. In Japan, about 70% of patients with diabetic kidney disease were referred to nephrology at an advanced stage of CKD (stage 4 or 5) [55].

Patient Awareness

CKD is mostly a silent disease, often progressing without noticeable symptoms in its early stages [56]. Even after being referred to a nephrologist, some patients may underestimate their condition and fail to adhere to medical advice [57-59]. A survey conducted among CKD patients showed that 64% of patients did not have any knowledge of their CKD status. Almost one in every five CKD patients (22%) was not informed of the reason for referral to nephrology specialists, and many patients did not receive predialysis education. Non-awareness of the existence of renal disorders was linked to late referral to nephrology specialists. This could be a result of the lack of regular screening among patients with a high incidence of developing CKD, such as hypertensive or diabetic patients [11,32].

Physician-related factors

Demographic factors overlap between patient-related and physician-related factors, as referral decisions in specific populations often depend more on physician perspectives than on actual differences in patient behavior or disease presentation. Major physician-related factors include lack of knowledge about CKD referral criteria, misinterpretation of CKD staging, and over-reliance on serum creatinine as the sole indicator of kidney function.

Lack of Knowledge of Referral Indications

Renal diseases, due to their often insidious and complex presentation, can be challenging for non-nephrologists to recognize. In addition, physicians may be unaware of the indications and benefits of early referral. An online questionnaire survey was conducted on 497 internal medicine residents in the US to determine their perception of the indications for referral in CKD patients [60]. The majority correctly identified a rapid decline in kidney function as a reason for referral, but failed to identify most of the other reasons for referral, such as proteinuria, anemia, and hyperkalemia [60]. In another survey, 90% of referring primary care physicians felt they had inadequate training in the timing of referrals and the indications for referrals [47]. Among them, family physicians were more likely to refer earlier than internal medicine specialists [47]. Wang reported that under-perception of CKD among primary care physicians (PCPs), rather than unsatisfactory health-seeking behavior or low detection rates, was the main cause of under-diagnosis of CKD in China [61].

Lack of Standardized Screening Tools

One of the pitfalls in explaining poor knowledge is the common dependence on serum creatinine as a kidney disease marker, which is affected by multiple variables, including diet type and the patient’s muscle mass [62]. Several calculations were introduced, using serum creatinine as one factor, to develop the estimated glomerular filtration rate (eGFR)-based reporting system [63]. Buttigieg et al. conducted a retrospective analysis of CKD referral practices among non-nephrology specialists. They reported that urine tests remain largely underutilized and only a minority (16.4%) of patients with an eGFR <30 mL/min/1.73 m² were referred to a nephrology specialist [52]. An Australian study audited the referral trends to nephrology using a four-variable Modification of Diet in Renal Disease (MDRD) formula, which was inserted into the results with every order for serum creatinine. There was a noticeable 40% increase in the referrals to nephrology monthly, most of which were for older individuals, those with diabetes, and individuals with CKD stage 3 [64]. A study by Choukem et al. observed that 41-44% of non-nephrology specialists were unaware of the definition of CKD or knew that CKD had five stages. Twelve percent of these physicians solely used serum creatinine for diagnosis [65].

Health system-related factors

The health system plays a major role in either facilitating or impeding referrals of CKD patients. Managed care, reimbursement rates, and scarcity of dialysis centers in certain areas are considered hindering factors [10]. Different system-related factors play a role in referral rate, including the lack of organized referral pathways, different referral criteria across guidelines, limited access to specialists in remote areas, and insurance barriers [14,20,40].

Lack of Referral Pathways

A key challenge in the referral process is the lack of referral pathways, which leads to inconsistencies in how and when patients are referred to nephrologists. Additionally, limited access to specialists, particularly in underserved or rural areas, further exacerbates these delays [66]. This is compounded by missed early diagnoses, where inadequate screening and late recognition of CKD result in late referrals and poorer patient outcomes.

Limited Access to Specialists

The scarcity of nephrologists compared to other specialties, long waiting times, and poor feedback loop with PCPs were also reported in the literature [10]. Recently, telemedicine-based nephrology models and virtual triage systems enhanced referral pathways, reduced waiting times, and supported earlier specialist input in both urban and rural settings. Several digital tools were integrated into primary care, including electronic alerts and risk calculators, which facilitated earlier identification of high-risk CKD patients and more timely nephrology referral. A previous PCP survey highlighted limited access to nephrologists, where a nephrology consultation could take several months due to the scarcity of nephrologists [66]. Applying telemedicine in nephrological consultation could be a potential solution. A study from Chile demonstrated that telemedicine nephrology consultation reduced waiting time from 289 days to 23 days within a time frame of six months [67]. This significant reduction in waiting time could aid in the early referral of CKD patients and provide them with proper management. Healthcare systems need to balance between the demand for nephrology consultation and their available nephrology resources of specialists and other healthcare staff.

Insurance and Coverage Barriers

Insurance and coverage barriers significantly impact referral patterns and access to nephrology care. An evaluation of the United States Renal Data System (USRDS) was conducted, comparing around 8,000 patients with lupus nephritis who progressed to ESRD across insurance types and socioeconomic status [68]. The study demonstrated that patients with private medical insurance were older at the onset of ESRD compared to patients with Medicaid or no insurance [68]. In addition, the type of insurance correlated more with the age of onset of ESRD than with the socioeconomic status [68]. Okaka et al. reported that lack of insurance was the most frequent reason given by participants with referral delay [40].

Poor Coordination Between PCPs and Nephrologists

Poor coordination between PCPs and nephrologists has been consistently highlighted in the literature as a barrier to early referral. A US survey conducted among PCPs reported a lack of proper communication with nephrologists. In addition, an unclear alignment of roles and responsibilities between PCPs and nephrologists could create a potential delay in referral and decision-making [66]. The absence of comprehensive and timely communication and referral could impact the effective co-management of CKD patients.

Collectively, these patient-, physician-, and health system-level barriers contribute to delayed nephrology referral and a higher likelihood of patients presenting in advanced CKD stages or at the time of dialysis initiation. Such referral delays not only affect clinical outcomes but also have substantial economic consequences.

Financial burden of CKD

Cost and Resource Utilization in Early vs. Late Referral

Impact on clinical outcomes: Early referral of CKD patients to nephrology specialists positively impacts both clinical outcomes and resource utilization. A meta-analysis, including 630,000 CKD patients, observed that early referral was associated with a 33% lower mortality (HR=0.67; 95% CI: 0.62-0.72), long-term survival (HR=0.67, 95% CI: 0.60-0.74), and fewer hospitalizations, and better preparedness for dialysis with less incidence of emergency dialysis [32]. These clinical outcomes have significant benefits for short- and long-term expenditures. However, these pooled estimates should be interpreted cautiously due to clinical and methodological heterogeneity across studies, including variations in definitions of late referral, baseline CKD stage, and healthcare systems. Lee et al. found that early referral imposed a lesser financial burden on the healthcare systems before RRT initiation, where there was an approximate 27.92% cost reduction with early vs. late referral ($6,206±5,873 vs. $8,610±7,820, p<0.001) [26]. In Germany, a database of health claims from 80 insurance companies included 105,219 patients with CKD. Annual hospital admission rates per patient were significantly higher with late referral to nephrology specialists compared to earlier referral. The total costs per patient per year, including hospital care, ambulatory services, and medications, were significantly lower with early referral across all CKD stages (p<0.03). The same study showed that early referral was associated with stabilization of CKD stage, delayed onset of dialysis, and improved survival compared with late referral [29].

Impact of CKD progression: The only economic model assessing the cost-effectiveness of early referral was developed and published from the NHS perspective [69]. In this study, a Markov model was built based on the five stages of CKD and the levels of proteinuria, which are also markers of kidney disease. The model was run over a 35-year time horizon and used a discount rate of 3.5% for future costs and consequences. The incremental cost-effectiveness ratio (ICER) was £3,751 per QALY for stage 3a, £3,857 per QALY for stage 3b, and £5,923 per QALY for stage 4. Although this model indicates that earlier referral is superior due to higher care costs as the disease progresses, sensitivity analysis showed that the ICER was highly sensitive to changes in the baseline risks of CKD progression. Applying more conservative progression rates increased the ICER for early referral at CKD stage 3a to above £30,000 per QALY, which is the threshold used by the NHS for cost-effectiveness analysis [69]. Despite these results, it is important to note that referral rates varied depending on the criteria used. A study reported that the DEGAM guidelines resulted in a 4.9% referral rate, whereas the DGfN/DGIM criteria led to a higher rate of 8.3%. Additionally, the estimated costs of implementing these referral criteria were different when using different referral criteria. Therefore, early referral to nephrology specialists is expected to improve patient survival, optimize healthcare utilization, and reduce economic burdens by shifting from high costs of acute care to more cost-effective planned care.

Increasing Financial Burden of CKD With Progression

The burden and cost of CKD differ among countries, healthcare systems, reimbursement strategies, and insurance policies. Multiple studies have reported increased costs of care across advanced stages of CKD and have attempted to identify the underlying drivers.

Cost burden of renal replacement therapy: RRT costs have been growing at a rate of 6-12% per year over the past few decades in most of the developed countries [70]. RRT had the highest impact on hospitalization costs, and kidney transplantation offered substantially lower costs than dialysis over the years [71]. RRT, including dialysis or transplantation, accounts for 2-3% of the annual healthcare budgets of some health systems [63]. An Italian study observed that the annual healthcare cost of an average adult is approximately 10% of the cost of a patient on dialysis [72]. Nisar et al. reported that hemodialysis represented the major cost burden, with an average annual expense of $23,358 per patient. Whereas disease management was associated with a significantly lower average cost of $4,977 per patient per year [73]. Data from the UK Renal Registry identified the healthcare resources utilized in patients receiving RRT, including hemodialysis, peritoneal dialysis, and kidney transplantation. Multivariate regression analysis showed that the hospital cost for the first year of treatment was high across the three modalities. However, transplant patients had a lower annual cost compared to hemodialysis patients [74]. The modified trajectory of ESRD patients by altering the choice of RRT compared to standard practice was studied in France from a health insurance perspective. Standard practice is established worldwide to be hospital-based hemodialysis, which is associated with the highest fees. The authors concluded that shifting to other clinically beneficial modalities, such as peritoneal dialysis and home unassisted hemodialysis, was associated with reduced outpatient costs, except for daily home hemodialysis [75]. They highlighted the importance of the nephrologists' role in the decision-making process and the need for early information and a structured organization for adequate patient education [75]. Those findings reinforce the need for early referral to nephrology to allow adequate planning and preparation regarding the choice of renal replacement modality. Kidney transplantation also incurred substantial costs, with $75,326 for the first year and $16,672 annually thereafter.

Cost burden of chronic kidney disease stages: A previous review across 31 countries observed that the cost of CKD management escalates significantly as the disease progresses, with expenses increasing nearly fourfold from CKD stage 3a to 5. Annual costs per patient rise drastically from early CKD stages ($3,060) to dialysis ($57,334 for hemodialysis, $49,490 for peritoneal dialysis). In 2023, the total expenditure of the National Health Service (NHS) on renal care was estimated at £6.4 billion. It represented around 3.2% of the total spending of the NHS that year. These costs accounted for the direct cost of care, including inpatient, outpatient, and RRTs. Indirect costs included increased associated strokes, heart disease, and infections [76]. A secondary analysis of the Study of Heart and Renal Protection (SHARP) trial assessed the impact of CKD stage and cardiovascular disease on annual hospital costs [71]. The mean hospital cost per person-year was £1,055 for CKD stages 1-3 and £3,694 for CKD stage 4. It increased to £13,000 at CKD stage 5 without dialysis and to £20,511 as soon as dialysis was initiated. Figures from the United States show an exponential increase in costs with increasing CKD stages for patients covered by Medicare and commercial insurance, even at early stages. Inpatient costs were the key drivers of this cost increase, mostly due to higher 30-day readmissions that increased steadily with each CKD stage [77].

Cost burden of CKD complications and comorbidities: Complications of late CKD stages significantly increase the financial burden [71]. The cost of comorbidities also added a significant burden on healthcare systems. Diabetic patients with severe nephropathy had 20.9 inpatient events over two years compared to 1.1 events for matched diabetic patients without nephropathy. Their cost of care was seven times that of patients with diabetes but without recorded nephropathies [78]. Despite those numbers and the understanding that early targeted intervention improves outcomes and lowers resource use, the cost-effectiveness of early referral to nephrology has not been extensively studied. Managing CKD complications is also costly, with annual expenses per patient estimated at $18,294 for myocardial infarction, $8,463 for heart failure, $10,168 for stroke, and $5,975 for acute kidney injury [79].

Proposed implications

Developing health policies requires the identification of reliable factors of increased CKD costs, one of which seems to be the timing of referral to specialized nephrology care. In-depth understanding of the factors responsible for late referral provides a description of the determinants of healthcare utilization and should be looked at from a variety of perspectives. Several proposed recommendations can be inferred from this review.

Unification of Referral Guidelines

Various clinical practice guidelines exist internationally to guide the management of CKD; however, each society has different referral criteria [20]. This review highlighted that different referral criteria result in different referral rates and different cost estimates. A study conducted at Brigham and Women’s Hospital in Boston projected the volume increase in their nephrology outpatient clinics if the KDIGO guidelines for referral were strictly followed. This large increase in the number of referrals uncovered a supply-demand mismatch that made the application of these guidelines particularly challenging [63]. Unifying international practice guidelines, using an age-adapted outlook on this chronic disease, will allow the establishment of clear referral criteria that are cost-effective for most health systems.

Developing Risk-Based Tools for the Evaluation of Kidney Function

A NICE recommendation exists to base the referral of patients to nephrology services on the Kidney Failure Risk Equation (KFRE) rather than on the eGFR level alone. The KFRE allows the identification of an individual’s risk of accelerated renal failure, and a five-year cut-off risk >5% is an indication for referral. Implementation of this method allowed GPs to identify the proportion of individuals at elevated and unrecognized risk who would benefit from early referral to nephrology specialists [80]. In the Canadian province of Manitoba, utilizing the KFRE in the triage process of new nephrology patients was found to be an effective health policy tool for reducing the waiting time and improving access to care for a population that is at the highest risk of kidney failure [81]. The global implementation of this strategy would allow the standardization of referrals and the appropriate identification of suitable patients who are at high risk of progressing to renal failure.

Utilizing Electronic Medical Records (EMRs) for Referral Identification

PCPs should be aware of referral criteria and the available tools for diagnosing and managing different CKD stages. Inconsistencies in defining early referral should be addressed, and effective policies must be developed to reduce late referrals [82]. Electronic Medical Records (EMRs) could be utilized to alert physicians about patients’ risks of CKD and prompt referral. Several studies were conducted to assess the success of those interventions. A US study granted nephrologists EMR access to CKD patients' records and allowed them to initiate referrals when certain criteria were met. This allowed patients at the highest risk of progression to be referred, and those at low risk to be referred back to primary care [83]. Another study in the United Kingdom, using a networked EMR, enabled co-management of low-risk renal cases between a nephrologist and a GP, without the need for hospital referral [83].

Financial Incentives for GPs for Early Identification and Referral

The awareness of referral criteria and proper management of CKDs could be enhanced by modifying incentive strategies to promote appropriate referrals. Although financial incentives could influence physicians’ behavior in general, there are no studies assessing the impact of an incentive system in motivating PCPs to refer appropriate patients at an early stage to nephrology care. It remains one of the unmet needs.

Encouraging Screening Programs for At-Risk Populations

Guidelines for screening high-risk populations should be developed and implemented by healthcare systems [84]. Additionally, telemedicine should be integrated for CKD monitoring and timely referral to nephrology specialists in remote and rural areas [85,86].

Utilizing Telemedicine for Early Detection and Referral

Integrating telemedicine-based nephrology models and digital screening tools into routine primary care as enablers of earlier referral. Virtual consultation platforms, EMR-based alerts, and automated risk stratification algorithms can help primary care teams identify patients at high risk of progression and initiate early referral to nephrology specialists before the onset of advanced CKD or emergency dialysis. 

Limitations

A limitation of this review is that the societal costs of CKDs, including patients and their caregivers, missed days of work, lost job opportunities, and other costs, have not been assessed. This is a major factor that plays a role in further driving up the costs associated with this disease. Given the narrative design and heterogeneity in study outcomes, we did not apply a formal, standardized risk-of-bias assessment tool. Instead, we used a pragmatic appraisal approach, giving greater interpretive weight to larger, more recent, and methodologically robust studies. Finally, the types of studies referenced here include many prospective or retrospective chart reviews as well as surveys that don’t represent the highest level of evidence.

## Conclusions

In conclusion, the costs associated with CKD are substantial and are projected to increase with time. Early identification and appropriate timely referral of patients at high risk of kidney disease progression are important determinants of healthcare utilization in nephrology. The survival of CKD patients, their quality of life, and their hospitalization rates are greatly improved when proper specialized care is initiated early. This can also be an opportunity to avert increased costs. Adequate management of patients with CKD, aiming for reduced mortality, disease stabilization, delaying or avoiding dialysis onset, and choosing the most cost-effective modality for renal replacement therapy are all priorities that should be reinforced as they allow better allocation of scarce resources.
